# Knowledge, Attitude and Advice-Giving Behaviour of Community Pharmacists Regarding Topical Corticosteroids

**DOI:** 10.3390/pharmacy5030041

**Published:** 2017-07-25

**Authors:** Wing Man Lau, Parastou Donyai

**Affiliations:** Reading School of Pharmacy, University of Reading, PO Box 226, Whiteknights, Reading RG6 6AP, UK; p.donyai@reading.ac.uk

**Keywords:** topical corticosteroids, community pharmacist, attitudes, patient counselling

## Abstract

This study examines the relationship between community pharmacists’ knowledge, attitudes to information provision and self-reported counselling behaviours in relation to topical corticosteroids and adjunct therapy in atopic eczema. A mixed-methods approach was used whereby data from interviews with community pharmacists were used to design a structured questionnaire that a larger sample of community pharmacists completed anonymously. The questionnaire was completed and returned by 105 pharmacists (36% response rate). Pharmacists showed gaps in their knowledge on the use of topical corticosteroids in atopic eczema but had good understanding on the use of emollients. There was a significant correlation between pharmacists’ attitudes to information provision and their self-reported counselling behaviour for most themes except in relation to corticosteroid safety where less advice was given. Improving attitudes to information provision should correlate with increased counselling behaviour. However, for the theme of corticosteroid safety, further studies are needed to examine why in practice pharmacists are not providing patient counselling on this topic even though most agreed this is a topic patients should know about.

## 1. Introduction

Atopic eczema, synonymous with atopic dermatitis, is a common dermatological condition and in the top 50 of the most prevalent diseases worldwide [1]. Management of atopic eczema takes place predominantly in primary care and topical corticosteroids are widely prescribed to provide symptomatic relief of this condition. The safety and efficacy of topical corticosteroids are well established, provided that these products are used according to guidelines. However, in reality, some patients, and perhaps even some prescribers and other healthcare professionals, still feel apprehensive about the safety of topical corticosteroids even when used appropriately [2]. For example, patients or carers worry about skin thinning and systemic absorption leading to effects on growth and development. This situation remains, despite considerable efforts over many years by clinicians and researchers to highlight the therapeutic value and safety of topical corticosteroids when used correctly [3,4].

Fear and anxiety about applying topical corticosteroids is perhaps understandable if one considers the following contextual factors. Prescribing in dermatology can be seen as imprecise because often neither the patient nor the prescriber is certain about how much topical treatment to apply [5]. Yet, patients rely heavily on the directions of their prescribers and the product literature in an effort to use topical corticosteroids correctly. The problem is compounded by the fact that most dispensed topical corticosteroid products carry labels that read ‘use as directed’ and ‘apply thinly’ as a norm. This in itself presents problems, as such instructions are subjective and ambiguous but, in the meantime, carry the connotation of being hazardous if not adhered to [6,7]. 

A number of studies have clearly shown that patients are not sufficiently advised by their healthcare professionals on topical corticosteroids and their use [8,9,10,11,12]. Current evidence also indicates that the lack of patient education on topical corticosteroid use has had a negative impact on the treatment they receive [12,13]. Unjustified fears of topical corticosteroids interfere with patients’ adherence to treatment and potentially affect the treatment outcome. Such fears can potentially be overcome with effective patient education on topical corticosteroids, especially if targeted at changing core beliefs about topical corticosteroids [12,14]. This is a role that can be carried out by healthcare professionals, including community pharmacists.

Community pharmacists are well placed to ensure that patients have been adequately educated on their topical corticosteroids before treatment begins as they are often the health professionals that see the patient immediately before medication is provided to them [15]. This role is doubly important considering that education about topical corticosteroid treatment is thought to be lacking from prescribers and manufacturers [8,10]. Even if the message regarding the correct application of topical treatments has been given by doctors and nurses, the pharmacist has an important role in reinforcing this message and ensuring that patients remember and understand what they have been told [16]. It is also important to consider that some topical corticosteroids are available without prescription to self-medicating patients who may not have been seen by a prescriber at all. In such cases, the community pharmacist may be the only healthcare professional to have the opportunity to offer any patient counselling on topical corticosteroid use. 

Appropriate educational intervention by pharmacists has been shown to effectively improve medication adherence [15,17,18,19]. This provides a strong rationale for advocating that community pharmacists play an active role in terms of educating patients who use topical corticosteroids, especially considering that adherence has been traditionally low in dermatology [20]. Recently, a number of studies have investigated the role, personal views and diagnostic ability of the community pharmacist in dermatological conditions [16,21,22] and their level of confidence in relation to topical corticosteroids [23]. However, little is known about the relationship between the community pharmacist’s own knowledge and attitude towards information provision and their patient counselling behaviour. To investigate this important relationship, we designed, validated and conducted a survey that was informed by interviews, to examine community pharmacists’ knowledge, attitude towards information provision and self-reported patient counselling behaviour in relation to topical corticosteroids and adjunct therapy for the treatment of atopic eczema.

## 2. Materials and Methods

The study employed a sequential mixed-methods approach where face-to-face semi-structured interviews informed the design of a questionnaire. The questionnaire was validated and piloted before being sent to community pharmacists for completion and return. The study was approved by the University of Reading Research Ethics Committee (study number 15/12).

### 2.1. Semi-Structured Interviews

**Figure 1:**
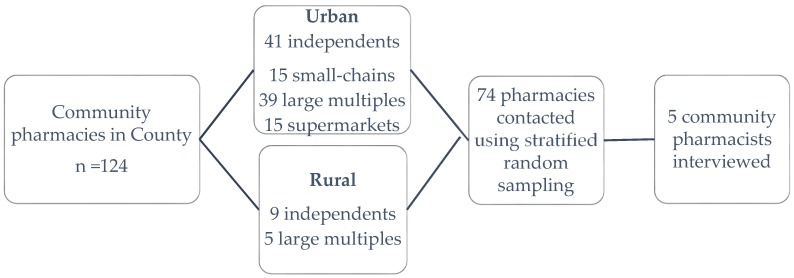
Stratified random sampling strategy based on community pharmacies in one English county. A total of 74 out of 124 community pharmacies in six different business type/location combination were contacted resulting in five responses.

Stratified random sampling was used to invite pharmacists working in a range of settings to interview using addresses listed in a public NHS database of pharmacies for Berkshire (one English county) (Figure 1). A total of 74 participant invitation letters were posted, with 12 or 13 going to each business type/location combination. The letter was addressed to ‘the pharmacist’ and invited them to a face-to-face interview about their views and ideas on the use of topical corticosteroids. A reminder letter was sent to pharmacies that had not responded after a month. Face-to-face, semi-structured interviews were conducted with the five community pharmacists (representing rural independent, urban independent and urban large multiple pharmacies) working in one English county who responded to the invitations. The interviewees each had between 13 and 34 years of community pharmacy experience. Each face-to-face interview was conducted in the setting of the interviewees’ pharmacy and lasted approximately 20 min.

Four pre-formulated vignettes were designed and validated during a preliminary phase, with a consortium of four pharmacists based at the School of Pharmacy (SOP). The vignettes were followed by a fixed number of questions that explored ideas about the cases further. Each vignette focussed on a different scenario likely to be encountered in a community pharmacy relating to skin conditions requiring a topical corticosteroid: (1) dispensing a topical corticosteroid prescription for an infant; (2) receiving a prescription for a potent topical corticosteroid; (3) addressing a customer’s request for an over-the-counter (OTC) topical corticosteroid based on a General Practitioner’s (GP’s) advice; (4) advice sought by a customer in relation to OTC topical corticosteroids (Table 1). In the questions that followed, participants were invited to comment on the cases in detail. 

Interviews were audio-recorded, transcribed verbatim but anonymised and then analysed using thematic analysis. Major themes arising from the analyses were subsequently used for informing the design of the main questionnaire.

### 2.2. Questionnaire Design

A questionnaire was designed to explore knowledge, attitudes to information provision, and frequency of self-reported counselling behaviours of pharmacists in relation to the use of topical corticosteroids and adjunct therapy in atopic eczema within a community pharmacy setting. The relevant National Institute for Health and Care Excellence (NICE) guideline [24] and the British National Formulary (BNF) [25] were used to devise 19 factual questions on the correct treatment of atopic eczema covering the five themes derived from the interviews. The five themes were: *non-pharmacological therapy*, indication for use of topical corticosteroid (*topical corticosteroid indication*), safety/effectiveness of topical corticosteroid (*topical corticosteroid safety*), application/instructions of topical corticosteroid (*topical corticosteroid application*) and *topical corticosteroid formulations* (detailed in the results section). Matching attitude (towards providing patients with information related to the above 19 facts) and counselling behaviour (frequency of providing this information in practice) questions were then devised with the help of five SOP pharmacists who reviewed the questions and provided feedback.

A web-based survey was developed and formatted using Bristol Online Survey consisting of four sections: Section A: 19 factual (knowledge) questions with fixed-response of Yes, No or Don’t know options; Section B: 19 matching attitude questions presented in a random order with a five-point Likert response scales from strongly agree to strongly disagree; Section C: 19 matching counselling behaviour questions enquiring about frequency of advice provided with five-point Likert response scales ranging from never to always; and Section D: pharmacists’ demographics relating to gender, age, work responsibility and details of any additional training on dermatology treatments. The questionnaire was formatted so that participants had to answer all questions within a section before the next section appeared and once they had submitted one section, they were unable to return to the previous section to change their answers. The online questionnaire was tested again with two pharmacists within the SOP to assess face and content validity.

The internet link to the online questionnaire, a cover letter and a participant information letter were emailed to all 294 community pharmacists working within one medium-sized chain of community pharmacies, with branches covering mainly the South East, Central and West of England but with branches also in the North of England, in August 2015. This one pharmacy chain was targeted for expediency—through an established teacher-practitioner relationship with the chain, we were able to gain the support and approval of the superintendent pharmacist for the work to go ahead, learn about the total number of pharmacists employed by the chain (to calculate the response rate) and to email all the pharmacists (via a secondary contact). A reminder email was sent to all the pharmacists on 1 September 2015 and the data collection stopped on 30 September 2015. Data were extracted directly from the Bristol Online Survey into the Statistical Package for the Social Science (SPSS) (Version 21.0. Armonk, NY: IBM Corp.) and analysed anonymously. Expert statistical advice was provided by Reading Statistical Services, which involved a statistician examining the questionnaire and the data generated extensively to advise on the suitable choice of analysis. Spearman rank correlation coefficient was used to evaluate the associations between attitude to information provision and self-reported counselling behaviour with the accepted level of statistical significance being *p* < 0.05.

## 3. Results

### 3.1. Interview Findings

Five main themes were derived from the interviews in relation to the advice-giving role of community pharmacists on symptoms and treatment of skin conditions relating to the use of topical corticosteroids and adjunct treatment. The identified themes and some supportive quotes are presented in Table 2.

### 3.2. Questionnaire Findings

#### 3.2.1. Study Population

Of the 294 pharmacists who were emailed the online survey link, 113 responses were received with 105 being useable. This resulted in a 35.7% response rate. Demographic details of participants are summarised in Table 3.

Only a third (36.2%) of the respondents had received some form of additional training on dermatology treatments after they had initially qualified as a pharmacist. Of those pharmacists, 28 indicated the details of training they had undertaken. Over half (57%) had used a Centre for Pharmacy Postgraduate Education (CPPE) programme as their source of training, 14% had received training through a drug company and the others carried out continuing professional development on this topic through local branch meetings, pharmacy conferences, presentations from dermatologists, or information in pharmacy journals. Nearly two thirds (61.9%) had carried out their additional training over 7 years prior to the survey. Managers made up 85% of respondents.

#### 3.2.2. Pharmacists’ Knowledge, Attitude to Information Provision and Self-Reported Frequency of Counselling Behaviours

Pharmacists’ accuracy of knowledge on the use of topical corticosteroids in atopic eczema is illustrated in Table 4. The majority of the pharmacists had a correct understanding of the use of emollients for atopic eczema and showed a good understanding of guideline recommendations on topical corticosteroid indications and application in atopic eczema. In terms of formulations, over 60% did not know how many topical corticosteroid potency categories exist (there are four) and nearly half of the pharmacists believed that the potency of a topical corticosteroid can be derived from examining the product packaging (which cannot be). The questionnaire results also indicated that pharmacists were less knowledgeable in terms of topical corticosteroid safety.

Pharmacists’ attitudes towards provision of information about topical corticosteroid and adjunct treatment are shown in Table 5 and their self-reported frequency of counselling behaviours are presented in Table 6. The statements listed in both tables are in the same order as the matching knowledge statements in Table 4 for the purpose of this manuscript but attitude and behaviour statements were randomly presented in the online questionnaires to avoid bias.

Our data showed that the majority of the pharmacists believed it is important that patients/carers have good understanding on all five themes in relation to the use of topical corticosteroids in atopic eczema, with 15 of the 19 statements demonstrating a mean attitude score of 4 or above (with 5 being the maximum); and more than 75% responded positively to nearly all of the attitude statements. However, only about half of the respondents believed it is necessary for the patients/carers to know that topical corticosteroid treatment should not be reserved for the worst areas in young children (i.e., can be used on all affected areas as indicated) and for the patients/carers to know that topical corticosteroid potency is not shown on product packaging.

The data presented in Table 6 show that the level of self-reported patient counselling activities in relation to topical corticosteroid treatment in atopic eczema was moderately high with a mean score between 3 and 4 for nearly all of the behaviour statements.

Counselling patients on the regular use of emollients in their atopic eczema treatment (82%) and informing them to apply emollients frequently (89%) were the most frequently reported counselling activities. One third of pharmacists indicated that they would frequently provide (often or always) counselling about the duration, frequency and quantity of the topical corticosteroid to be used as well as educating patients that topical corticosteroids help to reduce skin redness and itchiness. However, over 50% rarely provide counselling relating to topical corticosteroid formulations.

For the use and application of non-pharmacological therapy, nearly all pharmacists believed that patients should understand the importance of emollient application in order to improve atopic eczema and they self-reported a high frequency of counselling activities in this area. This was well reflected in the interviews where the importance of emollient and other non-pharmacological interventions were mentioned by all interviewees (Table 2).

The data from all 105 responses were analysed using two tailed Spearman’s correlations to examine the association between the mean score on ‘attitude’ and the mean score on ‘behaviour’ for each set of related questions within the 5 themes. For all the five themes, there was a positive relationship between attitude to information provision and frequency of self-reported counselling behaviour, but this was only statistically significant for four of the themes: *non-pharmacological therapy* (*r* = 0.483, *p* < 0.005), *topical corticosteroid indication* (*r* = 0.358, *p* < 0.005), *topical corticosteroid application* (*r* = 0.244, *p* = 0.012) and *topical corticosteroid formulations* (*r* = 0.265, *p* = 0.006). For the set of 4 questions that related to *topical corticosteroid safety/effectiveness*, the relationship was not statistically significant (*r* = 0.165, *p* = 0.093), meaning that although a large percentage of participants agreed that it was important to provide a range of advice about the safety of topical corticosteroids, they were less likely to provide this advice in practice.

## 4. Discussion

This is the first study that has used a mixed-methods approach to explore the counselling role of community pharmacists in the UK in relation to the use of topical corticosteroids in atopic eczema/atopic dermatitis. The face-to-face interviews provided insights about the types of knowledge pharmacists deemed important to communicate in topical corticosteroid related cases. Five main themes were derived and used to formulate a questionnaire to measure the general knowledge, attitude to information provision and self-reported frequency of counselling behaviour of a wider group of community pharmacists in relation to the use of topical corticosteroids and adjunct treatment. Community pharmacists had a good general understanding of most areas relating to topical corticosteroids and adjunct therapy in atopic eczema. In addition, they mostly agreed with statements about what patients should know/understand in line with current advice about the treatment of atopic eczema and indicated that they counselled patients about these during their practice. However they were less likely to provide patient counselling on the safety of topical corticosteroids, possibly due to their own lack of knowledge in this area. Less than 50% of the pharmacists correctly answered questions relating to skin thinning and use of topical corticosteroids in young children. This could be explained by the facts that less than 40% of the pharmacists in the current study had undertaken additional/postgraduate training in dermatological treatment and the majority of those who had, had carried out the training over 7 years ago. This number is lower than another UK study carried out by Tucker [16] where 65% had undertaken training; CPPE) training packages were reported to be the most commonly used sources with about 50% in both studies. 

Community pharmacists play a vital role in educating patients in the use of topical corticosteroids [15]. However, it is important that pharmacists themselves have the correct understanding before they pass on advice and knowledge to patients and carers. Research has shown that misleading information provided by pharmacists leads to a major impact on the perceptions of topical corticosteroids in the general public [26]. Charman et al. [12] found that 73% of patients or carers worried about using topical corticosteroids on their own or their child’s skin and 30% were unable to correctly classify the most commonly used topical corticosteroids. In the current study, over 60% of pharmacists had incorrect knowledge on the number of potency categories in existence and 75% believed that it is not important for the patients to know the potency of their topical corticosteroid. Also, only 44% understood that side effects are uncommon when topical corticosteroids are used appropriately and, interestingly, a similar result (44%) was reported in a study carried out in Australia reporting baseline pharmacist knowledge, which was significantly increased post-education to 89% [27].

Although the BNF, a drug formulary that all UK pharmacists refer to, has clearly indicated that ‘treatment should not necessarily be reserved to treat only the worst areas’, only 23% of the respondents in this study had the correct knowledge on this, which could explain why only 22% reported they would provide such advice routinely; yet more than half (54%) actually believed that it is important for patients or carers to know this same information. This suggests that increasing pharmacist knowledge or empowering them in other ways could potentially increase the provision of counselling in this area. This is also true in term of pharmacists’ knowledge about topical corticosteroid potency; 63% did not know the potency grading, but 75% believed it is still important for the patient to know, and despite this, 73% would routinely provide advice on topical corticosteroid potency. This raises the question that if the pharmacists themselves do not have the correct knowledge, they could misinform patients and lead to topical corticosteroid phobia. Our study has highlighted the need for evidence-based health literacy education for pharmacists in order to avoid patient misunderstanding of topical corticosteroids. A study in Australia on topical corticosteroid phobia also highlighted this issue [26].

In the UK, General Pharmaceutical Council standards for pharmacy professionals stipulate that all pharmacists should provide patient-centred care [28]. However, our study indicates that while community pharmacists believe they have a role in providing advice on topical corticosteroid use as well as non-drug management of atopic eczema, they expressed that medication counselling is difficult due to patients often being unable to speak to a pharmacist. For example, one participant stated: “*So it is up to us (the pharmacists) to pass on the message. But it depends if they get to speak to the pharmacist or not*.” Some felt a dilemma when selling OTC topical corticosteroid products especially when another healthcare professional suggests that the patient should buy a product not licensed for the particular indication. For example, one participant said: “*GPs telling people to buy topical steroid that I am not supposed to sell them for. Or nurses as well telling them to go to the pharmacy to buy it to put on your face. Or my child is 7 and the doctor told me to come and buy this. Cause they could make it very difficult for us*”.

Pharmacists in this study stated that they would frequently counsel the patient on the quantity of topical corticosteroid to be applied but a third did not use the finger-tip unit as a guide. This finding is also in-line with the interview findings, where only one out of the five interviewees used the finger-tip unit in their counselling, whereas other pharmacists articulated ambiguous or confusing advice on the amount to be used (Table 2). For topical corticosteroid formulations, fewer pharmacists believed this area is important for the patients to know and this was reflected in the section on self-reported counselling behaviours. In terms of topical corticosteroid safety and effectiveness, although nearly all the pharmacists had a positive attitude, they did not routinely provide counselling on this topic. This could be due to lack of opportunity to discuss this topic in a consultation. Due to time constraints, pharmacists may prefer to counsel patients or carers on application of topical corticosteroids and use of emollients than specifically on topical corticosteroid safety. Points regarding time and counselling opportunity also came out from the interviews; for example, one participant stated: “*by giving an MUR have more time. Usually be able to find out how they use their cream*”. Potentially, if time is not the limiting factor, i.e., pharmacist time could be freed up for counselling, then improving pharmacists’ attitudes towards information provision could potentially improve counselling behaviour on the use of topical corticosteroids in the treatment of atopic eczema, as generally there is a positive association between the two concepts.

The strengths of this study are that we have used the mixed-method approach in which the second phase of the study was partly developed from the first. The qualitative study helped us to generate themes to be tested in the questionnaire, but vice versa, findings from the questionnaires could also be explained through insights obtained from the interviews. Also, the study obtained responses anonymously with pharmacists representing different age groups, years in practice and both genders completing and returning the questionnaire. We acknowledge that this study does have some limitations. The fact that we only used one medium-sized pharmacy chain for the distribution of the questionnaire and given the relatively small sample and response rate, mainly from pharmacy managers, restricts the generalisability of these findings to the wider community pharmacy population. Also, we cannot be sure that participants did not take time out to research answers for the knowledge section and cannot prevent the inherent biases in self-reporting of behaviours in questionnaires (rather than actually measuring behaviours).

## 5. Conclusions

It is apparent that UK community pharmacists do have some knowledge gaps in terms of the use and safety of topical corticosteroids in atopic eczema. Although pharmacists have the right attitudes about what patients or carers should know regarding the use of topical corticosteroids, it is vital that pharmacists increase their knowledge and eliminate misconceptions in this area to avoid misinforming patients or carers, which could ultimately lead to or compound topical corticosteroid phobia and non-adherence. Since pharmacist attitudes about information provision showed significant correlations with counselling behaviours in the main, efforts to improve counselling behaviours could focus on addressing perceived attitudes towards the provision of patient education.

## Figures and Tables

**Table 1:** Summary of the four different vignettes used in the face-to-face semi-structured interviews.

Vignette	Description	Topical Corticosteroid	Patient Age	Origin of Query	Main Query
1	Based around a topical corticosteroid prescription for a baby, supplemented with information about baby’s nappy rash and mother worrying about using topical corticosteroid on baby	Hydrocortisone Cream 1%	Baby <1 year	Prescription	Topical corticosteroid use in infant
2	Based around a mid-50s male with monthly repeats of Eumovate 0.05% presenting with a new Rx for beclometasone dipropionate 0.025% cream and supplemented with information that the man worries 0.025% is weaker than 0.05% for his flare-up psoriasis patches	Beclomethasone dipropionate cream 0.025%	Mid-50s male	Prescription	Topical corticosteroid strength
3	Based around a young man asking for Eumovate cream to buy OTC for his hands, supplemented with information that GP had advised him to buy the product OTC	Eumovate cream 0.05%	Young male	OTC	Advice with OTC topical corticosteroid
4	Based around woman asking for pharmacist advice about her itchy dry hands, supplemented with information she wants ‘stronger cream’	N/A	Middle-aged female	OTC	Recommend OTC topical corticosteroid

**Table 2:** Themes and selected supportive quotes regarding pharmacists’ advice-giving role on skin conditions and relating to the use of topical corticosteroids and adjunct therapy in the community setting.

Themes	Supportive Quotes
Non-pharmacological therapy	“And sometimes you hear advice about let the baby not wear a nappy for as long as possible so life style counselling you can give them, as well as to do with the actual cream itself”
	“So I would advise her to probably have the emollient cream handy in a handbag or in a pocket so she could apply it all the time, as long as she is not handling the food”
	“If it is stress related then there are counselling points on how to manage his stress and how to relax”
Topical corticosteroid indication	“We need to know what it has been used for. How long it has been used”
	“I probably need to find out the indication, which part of the body he uses it for. And just find out from the patient first. I will check why, why the doctor prescriber this for him”
	“Important to explain what the purpose of this (topical corticosteroid) cream is”
Topical corticosteroid safety	“Advising him that after 7 days (of topical corticosteroid), if not better need to go back to the doctor”
	“I would explain to the mother not to worry about the hydrocortisone cream. It is very useful. Using this hydrocortisone cream won’t hurt, provided that she uses it sparingly as directed by the doctor”
	“I would quite often explain about the side effects of overuse, about thinning of the skin”
Topical corticosteroid application	“How many, how to apply it onto the sort of size area. So you need to say to the patient how it is applied”
	“Talk to him about how frequent to use it, and how to use it”
	“Use the finger tip method, just to explain how much cream to apply onto the area so she is using enough so that it is effective”
Topical corticosteroid formulations	“It is important as a pharmacist that you explain there are different groups (of topical corticosteroids)”
	“I would explain to him that the beclometasone is stronger than clobetasone, but the strength of beclometasone is lower than clobetason does not mean it is less stronger than clobetasone”
	“Part of the counselling will be to explain about not just the percentage but steroids are graded in terms of how strong they are”

**Table 3:** Survey respondent characteristics (*n* = 105).

Characteristic	*n (%)*
*Gender*	
Male	51 (48.6)
Female	54 (51.4)
*Age*	
20–30	30 (28.6)
31–40	31 (29.5)
41–50	20 (19.0)
51–60	21 (20.0)
61–70	2 (1.9)
>71	1 (1)
*Pharmacy Rolev*	
Manager	89 (84.8)
Employed pharmacist	16 (15.2)
*Number of Years in Community Pharmacy Practice*	
<5	26 (24.8)
5–10	24 (22.9)
11–15	17 (16.2)
16–20	15 (14.3)
21–25	4 (3.8)
25–30	8 (7.6)
>30	11 (10.5)
*Employment Type*	
Full-time	90 (85.7)
Part-time	15 (14.3)
Locum	0 (0)
*Additional Training on Dermatology Treatments*	
Yes	38 (36.2)
No	67 (63.8)

**Table 4:** Pharmacists’ knowledge toward topical corticosteroids and adjunct therapy in atopic eczema.

Statements	*n* (%) Correctly Answered
*Non-pharmacological therapy*	
Emollients are a first-line therapy for eczema/dermatitis	102 (97.1)
Many emollient products are available with no one product suiting all patients	99 (94.3)
A topical steroid can be applied on the same area as other topical preparations (including an emollient) at the same time. (NB: the correct response was ‘no’)	79 (75.2)
The moisturising effect of an emollient is long-lived (NB: the correct response was ‘no’)	80 (76.2)
*Topical corticosteroid indication*	
Topical corticosteroids are the first-line treatment for flare-ups of eczema/dermatitis	82 (78.1)
Topical corticosteroids have anti-inflammatory and immunosuppressive effects	81 (77.1)
Topical corticosteroids are used to cure eczema/dermatitis (NB: the correct response was ‘no’)	93 (88.6)
*Topical corticosteroid safety*	
The choice of topical corticosteroid potency depends on the severity and site of the condition	105 (100)
Absorption of topical corticosteroid is greatest where the skin is thick (NB: the correct response was ‘no’)	93 (88.6)
Side effects, such as skin thinning, are common even when topical corticosteroids are used appropriately (NB: the correct response was ‘no’)	46 (43.8)
Topical corticosteroids should only be used to treat the worst affected areas in young children (NB: the correct response was ‘no’)	23 (21.9)
*Topical corticosteroid application*	
In general, topical corticosteroids should be used for 7–14 consecutive days	79 (75.2)
Topical corticosteroids should always be applied exactly twice a day (NB: the correct response was ‘no’)	85 (81)
A sufficient quantity of topical corticosteroid should be applied to cover all affected areas	86 (81.9)
The quantity needed for each application can be measured using the finger-tip unit	95 (90.5)
If a topical corticosteroid is needed long term, a regular break in treatment should be incorporated	93 (88.6)
*Topical corticosteroid formulations*	
In the UK, topical corticosteroids are categorised into three potency grades (NB: the correct response was ‘no’)	39 (37.1)
Corticosteroids, androgens and oestrogens are steroid hormones with the same mechanism of action (NB: the correct response was ‘no’)	77 (73.3)
The potency of a topical corticosteroid can be worked out from the manufacturer product packaging (NB: the correct response was ‘no’)	62 (59)

**Table 5:** Pharmacists’ attitudes towards provision of information about topical corticosteroid treatment in atopic eczema using a five-point Likert response scale where 1 = strongly disagree to 5 = strongly agree.

Attitudes (It is Important That Patients Understand/Know…)	Strongly Agree/Agree *n* (%)	Neutral *n* (%)	Strongly Disagree/Disagree *n* (%)	Mean (SD)
**Non-pharmacological therapy**				
…emollients are beneficial in eczematous disorders	98 (93.3)	7 (6.7)	0	4.5 ± 0.6
…different emollient are available to suit individual preferences and needs	101 (96.2)	4 (3.8)	0	4.4 ± 0.6
…to leave a short interval between application of different topical preparations	99 (94.3)	6 (5.7)	0	4.4 ± 0.6
…the moisturising effect of an emollient is short-lived	86 (81.9)	8 (7.6)	11 (10.5)	4.1 ± 1.0
**Topical corticosteroid indication**				
…topical corticosteroids should only be used to control flare-ups	93 (88.6)	8 (7.6)	4 (3.9)	4.3 ± 0.8
… they have been prescribed a topical corticosteroid because they have a chronic inflammatory skin disorder characterised by itching, dry skin and redness	80 (76.2)	21 (20)	4 (3.9)	3.9 ± 0.78
…topical corticosteroids do not cure their skin conditions	93 (88.6)	9 (8.6)	3 (2.9)	4.2 ± 0.72
**Topical corticosteroid safety**				
…the potency of topical corticosteroid used is based on the severity and site of their skin condition	87 (82.9)	16 (15.2)	2 (2)	4.0 ± 0.7
…the thinner the skin, the higher the chance of topical corticosteroid side effects	96 (91.4)	8 (7.6)	1 (1)	4.3 ± 0.6
…side effects are rare if topical corticosteroids are used appropriately	84 (80)	13 (12.4)	8 (7.6)	4.0 ± 0.9
…treatment should not necessarily be reserved for the worst areas in young children	57 (54.2)	25 (23.8)	23 (21.9)	3.5 ± 1.1
**Topical corticosteroid application**				
… the duration of topical corticosteroid treatment	95 (90.5)	8 (7.6)	2 (1.9)	4.4 ± 0.7
… the frequency of topical corticosteroid application	96 (91.4)	7 (6.7)	2 (1.9)	4.4 ± 0.7
…the quantity of the topical corticosteroid to apply for each application	96 (91.4)	9 (8.6)	0	4.4 ± 0.7
…the finger-tip unit	92 (87.6)	12 (11.4)	1 (1)	4.4 ± 0.7
…topical corticosteroid should not be used long term without drug-free periods	92 (87.6)	10 (9.5)	3 (2.9)	4.3 ± 0.8
**Topical corticosteroid formulations**				
…the potency of their topical corticosteroid preparation	79 (75.2)	22 (21)	4 (3.9)	3.9 ± 0.8
…topical corticosteroids are not the same as steroids used in contraceptive pills or for bodybuilding	87 (82.9)	18 (17.1)	0	4.2 ± 0.7
…topical corticosteroid potency is not shown on product packaging	59 (56.1)	26 (24.8)	20 (19.1)	3.5 ± 1.0

**Table 6:** Pharmacist self-reported counselling behaviour in relation to topical corticosteroid treatment in atopic eczema with a five-point Likert response scale where 1 = never and 5 = always.

Self-Reported Behaviour (I Advise Patients/Carers…)	Never/Rarely *n* (%)	Sometimes *n* (%)	Often/Always *n* (%)	Mean (SD)
**Non-pharmacological therapy**				
… to also include regular emollient use in their treatment	4 (3.8)	15 (14.3)	86 (81.9)	4.18 ± 0.88
… to change to another emollient if patients have not gained relief from their current emollient	12 (11.4)	26 (24.8)	67 (63.8)	3.74 ± 0.99
…to leave a short interval between application of different topical preparations on the same area	18 (17.2)	27 (25.7)	60 (57.1)	3.65 ± 1.11
… to apply emollients frequently for continued moisturising effects	4 (3.8)	8 (7.6)	93 (88.6)	4.39 ± 0.82
**Topical corticosteroid indication**				
…that topical corticosteroids should be reserved for flare-ups	16 (15.2)	34 (32.4)	54 (52.4)	3.51 ± 1.09
… that topical corticosteroid helps to reduce skin redness and itchiness	8 (7.6)	27 (25.7)	70 (66.7)	3.92 ± 0.78
…topical corticosteroid can only provide symptomatic relief	33 (31.5)	37 (35.2)	35 (33.4)	3.02 ± 1.08
**Topical corticosteroid safety**				
… that topical corticosteroid potency selected is based on the severity and site of their condition	29 (27.6)	35 (33.3)	41 (39.1)	3.1 ± 1.07
… to look out for further skin thinning when a topical corticosteroid is to be applied to skin that is thin	37 (35.2)	25 (23.8)	43 (41)	3.02 ± 1.23
… that side effects are uncommon when topical corticosteroids are used as directed	25 (23.8)	48 (45.7)	32 (30.5)	3.04 ± 0.96
… that it is appropriate to treat all affected areas even for young children	43 (41.0)	39 (37.1)	23 (21.9)	2.7 ± 1.0
**Topical corticosteroid application**				
… about the duration of topical corticosteroid treatment	10 (9.5)	28 (26.7)	67 (63.8)	3.73 ± 0.94
… about the frequency of topical corticosteroid use verbally	5 (4.8)	24 (22.9)	76 (72.3)	3.98 ± 0.88
…about the quantity of topical corticosteroid needed for each application	10 (9.5)	29 (27.6)	66 (62.9)	3.7 ± 0.92
…about the finger-tip unit	34 (32.4)	33 (31.4)	38 (36.2)	3.11 ± 1.19
… that drug-free periods should be incorporated in their topical corticosteroid treatment plan if it is for long term use	29 (27.6)	39 (37.1)	37 (35.3)	3.11 ± 1.14
**Topical corticosteroid formulations**				
…on the potency of their topical corticosteroid preparations	28 (26.7)	33 (31.4)	44 (41.9)	3.17 ± 1.03
…that a topical corticosteroids is not the same as steroids used for contraception or bodybuilding	57 (54.3)	25 (23.8)	23 (21.9)	2.41 ± 1.2
…on topical corticosteroid potency by adding potency grade on the dispensing label	59 (56.2)	26 (24.8)	20 (19.1)	2.44 ± 1.37
